# The influence of front-of-pack nutritional labels on eating and purchasing behaviors: a narrative review of the literature

**DOI:** 10.1007/s40519-022-01507-2

**Published:** 2022-11-11

**Authors:** Claudia Penzavecchia, Patrizia Todisco, Luca Muzzioli, Andrea Poli, Franca Marangoni, Eleonora Poggiogalle, Anna Maria Giusti, Andrea Lenzi, Alessandro Pinto, Lorenzo Maria Donini

**Affiliations:** 1 Medical Pathophysiology, Food Science and Endocrinology Section, Food Science and Human Nutrition Research Unit, Experimental Medicine Department, Sapienza University, Ple. Aldo Moro, 5, 00185 Rome, Italy; 2 Eating Disorders Unit, Casa di Cura “Villa Margherita”, Arcugnano, Vicenza Italy; 3 NFI-Nutrition Foundation of Italy, Milan, Italy

**Keywords:** Front-of-pack nutrition label, Eating pattern, Eating disorders, Healthy diet, Positive nutrition

## Abstract

**Background:**

Front-of-Pack Nutritional Labels are considered a useful tool to help consumers orient themselves in their food choices and direct their behavior toward a healthier diet. FOPNL development and use are part of a framework that includes cognitive, biological, hedonic and cultural aspects, able to affect consumers' eating and purchasing behavior.

**Aim:**

Given the complexity of the matter, the aim of this narrative review is to analyze the combination of different factors that drive food choices and eating behaviors and to highlight some aspects that are not fully studied.

**Methods:**

The authors conducted the research using a top-down approach at first, followed by a bottom-up approach; starting with general considerations about the purchasing process, gradually narrowing the discussion to a specific sub-population, and finally extending the discussion back to more general reasonings about the direction to adopt in future, or at least to evaluate, for effective communication.

**Results:**

Biases and attitudes toward food products were found to regularly interfere with buying behavior patterns, making it impossible to standardize an average consumer. This reflects in current research, increasing the complexity of the topic. All determinants influencing food choices are often assessed individually rather than in a synergistic and multidimensional context, while the purchasing scenario is characterized by multiple stimuli to which the consumer is subjected. FOPNLs’ impact on perceived healthiness has been studied in different conditions, but some population subgroups have not been sufficiently represented. In particular, the effect of FOPNLs on consumers suffering from eating disorders is understudied and needs further attention. Furthermore, some approaches can be compared to “negative nutrition” or “loss-framed communication”, putting nutrients out of context, emphasizing losses more than gains and risking promoting negative feelings in consumers.

**Conclusion:**

Due to the heterogeneity of studies, evidence on what works best in driving people to adopt lasting lifestyle changes is still mixed. Science communicators and policymakers should consider the possibility that a multi-component approach incorporating nutrition information and education may be a key strategy to promote consumers’ self-consciousness and to support them in their cognitive efforts toward a healthy and sustainable diet.

**Level of evidence:**

Level V, narrative review.

## Introduction

Human nutrition represents a complex system in which, alongside biological aspects, other dimensions related to social and cultural values, to the economic impact on territories and people, to the type of response to sensorial stimuli and to the sustainability of the agri-food chain, all play a significant role. Humans do not eat to introduce nutrients, but for a number of complex reasons that combine nutritional and neuroendocrine aspects with local culture, the history of the food system, gastronomic traditions, and hedonistic and sensory aspects.

To help consumers orient themselves in the field of the food supply and to try to direct their behavior toward a healthier and more sustainable diet, nutritional labels (usually on the back of the package, or BOPLs) have been placed on food packaging. The primary objective of food labels is to inform consumers about the nutritional contribution of different foods to the overall composition of the diet, and it is achieved through a standardized list of nutrients contained in the food [1], optionally supplemented by a series of additional information.

Food labelling has therefore two main purposes: (1) to communicate complex information to consumers in a simple way and in a standardized format, to guide food choices and behaviors; (2) to stimulate the food industry to reformulate some products in a healthier sense [2].

To support the policies for the prevention of diet-related non-communicable diseases (NCDs), front-of-pack nutritional labels (FOPNLs) have been suggested as a useful tool: they provide a global assessment of the healthiness of a product in various forms. This strategy is proposed in association with other strategies that are partially applied in Europe, such as “reformulation agreements, restrictions on the marketing of foods rich of fats, salt and sugars, public procurement in favor of healthy food products, taxation of sugary drinks” [3].

FOPNLs can have different public health objectives (prohibitive, prescriptive or both) and their outcome results after considering nutrients considered “critical” (with negative value when consumed in excess, i.e., sodium, saturated and trans fatty acids—SFAs and TFAs—and sugars). Some FOPNLs “provide the percentage of energy and nutrients in relation to the standard requirement of 2000 kcal/day, while others provide ratings of the content (low, medium, high) of specific nutrients. Others calculate a score indicating the global quality of a product” [4]. They are sometimes assessed on a standard value of 100 g or 100 mL, but some refer to the portion available for consumption [4].

FOPNLs provide information with different complexity (they display nutrient specific information or a global judgment on the product) and “directionality”. They could be categorized as follows:

- Non-directive labels, providing information such as the name of the nutrient, the amount in grams, and the percentage of the total (e.g., Reference Intakes, NutrInform Battery)
- Semi-directive labels, not only providing nutritional information, but also judging the healthiness of the single nutrients using colors, words, or signs (e.g., the English traffic light or Multiple Traffic Light—MTL, Warning Signs which may feature the octagon “stop” or the words “rich in”).
- Directive labels, often aggregated in one symbol (e.g., Swedish Keyhole, Nutri-Score) and combining several criteria to evaluate the healthiness of the product, expressing judgments, opinions and/or recommendations in general, without providing specific information on single nutrients.

Warning signs, nutrition and health claims are not technically FOPNLs although, in part and in some circumstances, they can be used in studies that test FOPNLs [4].

FOPNLs, because of their intrinsic nature, limit their considerations to a few elements that could affect, mostly negatively, consumers’ health status. Information, condensed into a color or number, has sometimes been found to facilitate better food choices and improve the nutritional quality of the shopping basket [5, 6], but sometimes it has on the opposite and been shown to misguide consumers and induce an inaccurate assessment of the product's healthfulness, which could result in higher consumption of unhealthy food [7, 8].

According to the WHO, “FOPL systems must be underpinned by a specific nutrient profiling model” [9].

Several nutrient profiling (NP) systems have been developed so far. There are some inherent difficulties in establishing a gold standard for NPs, such as the lack of uniform data for the composition and consumption of foods across countries, the relative differences in nutrient intake recommendations and dietary guidelines, the different policy interventions between countries and the conflicting results undermining the authenticity of the single models [10].

These different results are the reason why most studies underscore the need for future investigations before making any health policy decision [11].

The approach of some FOPNLs can be assimilated to a "negative nutrition" or “loss-framed communication”: this communication focuses the attention on single/distinct foods or their nutrients, decontextualizing them from the complex system that leads consumers to make food choices and the whole community to develop the agri-food chain and even more from the concept of a healthy dietary pattern. The message could be one that underlines the losses more than the gains, eliciting negative emotions in the consumers [12]. Currently, research has focused primarily on efforts to promote the performance of a single intervention, while what seems to be needed is to motivate people to perform a series of behaviors, i.e., both the initiation and the maintenance of health practices [13]. Besides that, the effectiveness of message framing has a series of modulating factors (e.g., characteristics of the message recipient and of the desired behavior).

Moreover, to date, no study focused on the potential impact of FOPNLs on different psychological phenotypes, involving either people suffering from diagnosed and borderline eating disorder (ED), or subjects with other psychological eating-related conditions such as orthorexia nervosa (ON) [14].

The aim of this narrative review is to explore the combination of different factors that influence food choices and eating behaviors and to underline some aspects that are not yet thoroughly clarified in the extant literature in relation to FOPNLs, to encourage further research in the field, such as: the purchasing behavior patterns, the determinants of propensity to buy, the determinants that influence food choices, the possible relation with eating disorders, and, lastly, the communicative approaches in the field of nutrition. We searched the current literature on the potential impact of nutritional labels on disordered eating to understand how the behavior of population at risk of EDs is influenced by FOPNLs.

To outline the complexity of the issue and explain the difficulty in harmonizing FOPNLs with their intended objectives, this paper reviews the various, though not exhaustive, facets of the problem, starting with attitudes and factors that influence food choices (“Factors influencing purchasing behaviors”–“The determinants that influence food choices and consumers’ profiles”), continuing with possible implications on eating disorders (“Nutrition labeling and eating disorders”), and the use of different communicative approaches (“Negative and positive nutrition approach”).

## Methods

The authors started analyzing the purposes and possible effects of FOPNLs in two previous papers [4, 10]. These works led to some considerations about the gaps in the research done so far, i.e., the interaction between different determinants of food choices, the lack of focus on eating disorders and other population subgroups, the effectiveness of a message that focuses only on certain food features.

The authors then carried out their research in this narrative review starting from more general considerations on the purchasing process, gradually narrowing in on a particular sub-population, and finally extending again the discussion to more general considerations on the approach to be adopted, or at least evaluated, in future for an effective communication.

Therefore the authors, initially (“Factors influencing purchasing behaviors”), focused on the identification, not yet exhaustive, of several factors that may influence the consumer's decision at the time of purchase in general, and which may interact with the presence of a FOPNL. In this research, they identified some of the heuristics that may occur during the food choice.

Secondly, the authors focused on the hedonic and health aspects of food behavior (“Healthiness and hedonic aspects as determinants of propensity to buy”), which differentiate this type of behavior from others related to the purchase of other goods.

In the third thematic area (“The determinants that influence food choices and consumers’ profiles”), to emphasize the complexity of the phenomenon, the different determinants of food choices were identified: because of this great variety of factors, the population can not only be seen as a shapeless mass, but also as many different subgroups, each making their choices on the basis of different drives. Hence, the focus on a particular sub-population (“Nutrition labeling and eating disorders”), namely those at risk or suffering from eating disorders. Finally (“Negative and positive nutrition approach”), the research concluded with an analysis and a reflection on two contrasting types of communication, one based on positive messages and one on negative messages.

### Factors influencing purchasing behaviors

Purchasing behavior is the driving force behind selling processes. Psychologists and consumer behavior scientists have investigated why and how people purchase a certain product or why they show brand loyalty, for example. The combinations of the factors that influence the act of buying are countless and they generate different purchasing behaviors accordingly.

Therefore, the existence of an average “reasonably well-informed and reasonably attentive and circumspect consumer”, who is rational, consistent and pursues a personal “maximum utility”, is questioned by the existence of those several possible purchasing behaviors [15]. The existence of such a “universally reasoning consumer” [16] is therefore undermined by different attitudes toward the product (cognition, affect and conation), or “heuristics” approach (simple mental processes that are largely used to make quick decisions in stressful situations simplifying everyday life, but that can also lead to cognitive biases) These attitudes, as showed below, can also interplay with FOPNLs messages, leading to different results:

- *System 1 vs System 2*: this theory of thinking [17] assumes that people in real life usually rely on mental shortcuts to take decisions quickly (i.e., on System 1, which is considered simple, fast and impulsive), and only in circumstances of doubt or “discomfort” System 2 (thoughtful, effortful and slow) comes into play. In virtual settings, participants could artificially adopt System 2 because they have more time and their attention is explicitly called on the FOPNL, thus making an effort because they know they are expected to.
- *Loss aversion*: as the JRC points out, an example of behavior that could differ between life and virtual settings is the one of the “loss aversion” theory: people seem to consider losses as more important than benefits of equal magnitude: for example, [18] found that in their study consumers were more concerned with avoiding red colored labeled food than choosing green ones.
- *Rebound effect*: according to the JRC, it is a concept usually used in energy economics that describes a decrease in expected gains because of behavioral responses. In this specific case could lead consumers to increase the consumption of a food perceived as positive, “to an extent that offsets the objective of the label itself”. More or less like [19] point out when writing about environmental labeling of food “consumers that choose foods with lower CFs (i.e., carbon footprint) per mass will tend to spend less money on food, leaving them with more money to buy other products which may compensate the reduced food CF”.
- *Halo effect*: is a cognitive bias that leads to generalizing one's opinion of a given thing, on the basis of a single characteristic of the product: e.g., seeing the green label on a product could lead people who use this heuristic (more or less consciously) to automatically consider the product healthy tout court. Consumers could paradoxically no longer ask how much of it to consume and how often, because, if it is healthy, in their eyes, it means that it can be consumed ad libitum. Therefore, they could over-consume the product compared to the recommended portions and frequencies, with negative repercussions on energy, nutrients and the variety of foods [20, 21].
- *Optimism bias*: is an overconfidence bias that affects people who tend to overestimate their abilities and knowledge, claiming to know enough about something (e.g., FOPNLs) or to have understood how it works, even if it is not true. It seems that people are largely convinced that they do not need to change their diets [22]. This makes it difficult to perceive the need to have healthier habits, because these consumers mistakenly believe they are less at risk than others.

These attitudes and biases can interfere with the purchasing behavior and lead to different/distorted results either in virtual settings or in the real world, or they could be reinforced by FOPNLs, leading to paradoxical effects.

As the JRC points out [15], most of the studies analyze a consumer sample in a virtual steady-state model instead of a dynamic one, typical of real life. The abilities of food labels, and in particular of FOPNLs, are studied in isolated condition, mostly unaffected by external factors, with a potential overestimation of the benefits by overlooking confounding factors such as compensatory consumption, increased physical activity or consumption of foods perceived as more nutritious or healthy. In some studies, the ability of the consumers to understand and use the FOPNLs for their advantage is assessed by taking a snapshot of their understanding at a specific point of time, unaffected by advertising, informational and education programs [2]. The problem with these studies lies in the fact that it is possible that those heuristics cited above do not come into play when consumers find themselves in a virtual, guided version of reality.

In addition, the lack of data on nutrient intakes has often required the use of outdated consumer surveys, raising questions about the representativeness of the present population behavior [15].

A few studies have been carried out in real-world supermarkets, while the majority have been conducted in virtual or online settings with outcomes assessed immediately after a single exposure. Laboratory experiments may be very important, at least in the first phases, for controlling for confounding factors. On the one hand, they are artificial and overestimate effects (like magnifying glasses), on the other hand, they guarantee ceteris paribus conditions and thus causal effects.

However, field studies and real-world laboratories are needed to estimate the potential effects of health warnings (concerning food, alcohol, tobacco) since a real-life setting may give significantly different results due to important interfering and confounding factors [23, 24]. Findings from those studies suggest that FOPNLs or shelf labels may achieve a small degree of success at persuading shoppers to switch from less healthy to healthier foods (maximum 2.0% change) [25].

### Healthiness and hedonic aspects as determinants of propensity to buy

The propensity to buy could be driven by hedonic or healthiness aspects. Hedonic motives to buy food relate to features, such as taste, pleasure, or the search for experiences [26]. Healthiness is a less definable concept, but for some people healthy products could be the ones with lower energy density, while unhealthy products can be fast food, pizza, biscuits but also milk [21, 27, 28].

The use of color coding seems to increase the idea of healthiness when consumers are explicitly asked to choose a healthy product, but apparently this is not the case when they are asked to choose according to preference [29]. It should be remembered that healthiness is not a homogeneous concept, as it is often assumed in studies: it can mean different things to different people. For some, it is related to the amount of sugar, energy, or salt in a product, for others to the presence or absence of gluten, or to whether a product is organic or not, etc. [30]. Moreover, we should consider that the same concept changes within the same group over time (e.g., our attitude toward fats has changed over the last 50–60 years).

At the same time, it is theorized that one explanation for our behaviors is to find in implicit and explicit attitudes: the first ones are automatically activated responses to stimuli, while the second ones are rational behaviors formed after a deliberate thinking process. The two attitudes are not exclusive, we probably use both in many situations, leaning toward one or the other depending on the situation [26]**.**

The presence of attitudes that occur at an unconscious level might explain the dissonance between actual behavior (e.g., hedonic choices) and declared attitudes (e.g., healthy eating) in some consumers. Another important point to consider when we think about motives related to hedonism or healthiness, is the distinction between utilitarian and hedonic dimensions according to the nature of the product (or how we perceive it): the utilitarian dimension is probably not significant for products we link with pleasure, but it is significant for utilitarian-oriented foods, as it is described for organic foods [26]. Implicit attitudes could thus explain the predisposition to buy hedonic foods, and explicit attitudes the one to buy so-called utilitarian foods.

On the other hand, interestingly, a very recent study highlights the presence of some models that seem to suggest that there could be a symmetrically opposed mechanism to take into account: chronic consumption of hyper-palatable foods would lead to a shift in neurobehavioral processes toward hedonic processes regulating food intake and promoting hyperphagia [31].

The picture is still not clear enough to draw conclusions about the exact mechanisms behind purchases, and this gives us the measure of how difficult it is to understand and test consumer buying behavior unequivocally.

### The determinants that influence food choices and consumers’ profiles

While the key driver for eating is hunger, food choices can be determined by other factors, which are currently extensively studied with different results. Although works from different research fields (e.g., nutrition, psychology, social science, marketing) provided evidence with different perspectives, the factors affecting food choices can be leveled into three main groups, each including several categories [32]:

1. *Food-related features*: intrinsic features, such as color and aroma, and extrinsic features such as information and packaging;
2. *Individual difference*: biological (e.g., hunger, appetite, and taste), physical (e.g., access, skills of cooking, and time), psychological (e.g., mood and stress), cognitive (e.g., attitudes or preference, beliefs, and knowledge), and social (e.g., family, and peers) factors;
3. *Society-related features*: culture, economic variables, such as price and income, and policy.

Table 1 shows some categories and determinants influencing food choices [2, 22, 29, 33–39].

**Table 1:** Determinants of food choices and eating behavior (based on [2, 22, 29, 33–39])

*Biological*: hunger/satiety, appetite, sensory aspects, balance through the central nervous system, balance between macro-nutrients, energy density of the diet, volume of food or portion size, palatability related to sensory properties of the food (e.g., taste, smell, texture and appearance)
*Economic*: cost, income, availability
*Physical*: accessibility to shops, chronic or acute illness, skills (including cooking skills), time available (particularly felt as a barrier by young people and those with high levels of education)
*Social*: social class, culture, social context, ethnicity, religion, family, peer group, meal pattern (influenced by irregular life/working organization, snacking), social setting (considering food which is eaten outside home), institutional and geo-political system, habits/duties (cooking only for oneself or also for other people, particularly felt by women), social support, isolation
*Psychological*: mood and stress that can affect motivation (reduced or extreme concern about weight control), emotional context, personality traits and characteristics (e.g., impulsivity, novelty seeking or harm avoidance), perceived guilt, psychological frailty, depression, low self-efficacy
*Educational*: level of education, nutrition knowledge (both subjective and objective), sources of information and capacity to discriminate among these sources
*Others*: gender, age, retirement, unemployment, work shifts, student status, level of satisfaction with current diet, mentality (including the perceived lack of need to make dietary changes), attitude, esthetic ideals, biases, beliefs (including trust in the food system), influence of mass media, food marketing and advertising, sustainability, where one lives, the interaction of various determinants that can result in an “obesogenic” environment, perceived risk of food wastage leading to a reluctance to try ‘new’ foods for fear the family will reject them, environment

Therefore, alongside biological factors (i.e., hunger/satiety cues), there are other determinants which are shaped by the environment, such as individual knowledge, preferences, habits formed by past experience.

Some of these determinants usually interact and overlap at the moment of purchasing. For instance, the attitude and approval of the consumer toward a label is something to consider: a skeptical consumer will not be influenced to buy a product with a FOPNL that he/she does not trust [40]. In some studies, consumers were seen as willing to pay a little more if there was a logo/label (not necessarily a FOPNL) stating that the product was better than others, but at the same time the effect was lost if there were more logos on the packaging, or if the logo was not perceived as "trusted" [41].

Another example regards brand loyalty and taste preference which significantly influence consumers' level of understanding and willingness to pay and buy the product. While some studies highlight a change in buying behaviors correlated with the presence of a FOPNL, there seems to be no association with the healthfulness of the product: sometimes consumers do choose more products with a displayed FOPNL, but regardless of the nutritional characteristics of the product as communicated by the label itself [16].Furthermore, the ultimate purchase is often influenced by the expected taste (which is different from the taste per se). It is necessary to pay attention to the fact that products marked as “healthy”—i.e., green—are often perceived aprioristically as less tasty. This could theoretically lead to the paradoxical consequence of a lower purchase, as in some recent studies [42].

The sensory characteristics of food, the exposure to food, but also what can be learnt about flavor–nutrient associations and the resulting preferences are other important determinants of food choices.

Regarding individual determinants, the impact of food labels, and FOPNLs in particular, on perceived healthiness seems to be more pronounced in studies with a preponderant participation of female subjects, who tend to be more attentive to the concept of “healthy" [43]. It has to be noticed that nutrition labels are more likely to be read by those who have an interest in healthy eating**,** show better nutrition knowledge, and thus may already display healthier eating patterns [44]: in short, those who need it the least. On the contrary, it should be the less educated social classes who need an effective help to better guide or correct their eating behavior.

It could be observed that women are over-represented in the samples because they are predominantly the household food shoppers, but even so, the evolution of society is undermining this *status quo*, requiring all population subgroups to be equally represented in studies.

Similarly, the wealthiest social class tends to be influenced more than the disadvantaged ones by these labels. This can lead to situations where women from disadvantaged socio-economic classes seem to be more often affected by obesity than men and other women from middle and upper socio-economic classes, because they often buy from fast food outlets and opt for less fruit and vegetables, reporting making "unhealthy" food choices not because of their own preferences but because of those of the family members for whom they care [45].

In general, FOPNLs seem to be effective in changing consumer behavior if there is an "induced" consumer inclination (not only in health-conscious ones, but also in people with diseases) toward healthy choices, but also an optimal environment, and if these are supported by government and scientific authorities.

Data about the ability of FOPNLs in steering consumers purchases are therefore ambiguous and often self-reported feelings, the validity of which is to be questioned. For instance, it seems that personal relevance and the perceived need for information could be the main factor influencing both perceived healthiness and the intention to buy a product with a health claim [46].

Consumers are normally exposed to an information overload before and directly when at Point of Purchase, resulting in “a consumers’ confusion framework for healthy eating” [47], where it seems that people with low levels of knowledge and poor nutrition literacy (but sometimes high motivation e.g., dietary modification goals) could focus and make decisions only on the basis of information about 'key' nutrients (sugars, SFAs, salt, gluten), and not, for example, the whole dietary pattern or the whole food product, or irrespective of food’s caloric content [47, 48]. The Global Burden of Disease 2017 states that even if sodium, sugar, and fat have been the main focus of diet policy debate, the dietary risk factors for mortality (each accounting for more than 2% of global deaths) are not key nutrients but dietary patterns, such as diets high in sodium, low in whole grains, low in fruit, low in nuts and seeds, low in vegetables, and low in omega-3 fatty acids [49].

In addition, food choices could also be influenced by our natural tendency to overestimate our self-control and underestimate health risks, by the perceived product availability, by the biases we are subject to, and by hedonistic and reward processes (as previously mentioned in “Factors influencing purchasing behaviors” and “Healthiness and hedonic aspects as determinants of propensity to buy”), which are often stronger than homeostatic drivers.

The aforementioned determinants should be studied to better understand how a population respond to a stimulus giving the chance to profile and determine different consumers groups. Consumers’ profiling has the aim of providing detailed information on a target population, helping to gain in-depth insight into consumers’ sub-population with common behaviors. Thinking that students, workers, and shift workers, for example, respond to the same stimuli and make food decisions following the same patterns, might bring to a short-sighted view of the determinants that influence these and other types of choices. Forgetting that different groups exist when making decisions for the community should be avoided, as well as promoting tools designed only for specific categories of people, according to the food security definition. To date, we found no meta-analysis or systematic reviews that focused on consumers’ profiling on the basis of their response to FOPLNs. Only the FOPLNs effectiveness was assessed so far [11, 43].

Nevertheless, population groups are characterized by people who are very different from each other. A significant fraction of the working population habitually work on shifts (e.g., in Italy) [50], a population at risk of being overweight because it tends to consume snacks instead of full meals, more sugary drinks and less fruit and vegetables than recommended during night shifts [51]: this because of high consumption of food from vending machines or deliveries for several reasons (e.g., the canteen is too expensive or closed at night; preparing meals in advance at home means losing hours of sleep).

Another distinct population group is made up of university students: the reasons why students choose certain foods are related to stress, time, convenience and ease of preparing foods, beliefs about body image and self-control, and the palatability of foods. The most suitable type of intervention for them is probably the one aimed at strengthening their self-regulation skills, acting on their intrinsic motivations [52] and creating habits and knowledge that are stable over time, a goal that seems difficult to achieve with the mere affixing of a FOPNL on some products, especially considering the high risk that university students develop dysfunctional strategies around food as a coping reaction for stressful events and/or the transition from family to a new reality of independence.

Interventions in workplaces are interesting because of their ability to reach a wide audience (e.g., interventions in canteens, vending machines, nutrition education), especially if these activities are sustained over long periods.

Another interesting intervention setting are schools, because of their ability to reach students, their families, and teachers (e.g., using Internet and media to create awareness about food and to involve children in learning about, preparing and cooking the food they will eat).

Interventions in supermarkets are equally interesting and promising for increasing awareness and nutrition knowledge, but their long-term effect and effectiveness are unclear at present.

Nutrition education, however, seems to be more effective if it is combined with psychological and educational support to help make the behavior lasting and sustainable over time [53, 54].

The scarcity and conflicts of existing data come from the fact that some factors influencing food choices are not easy to measure and demonstrate, and some biological mechanisms are actually not yet understood. There is still a need for a complex model that better explains the determinants of food choices and enables knowledge to be translated into policy recommendations [34]. Therefore, at the moment, the results of the studies could be only stratified by the single determinants of food choice and the consumers’ subgroups on FOPLNs response are yet to be profiled.

### Nutrition labeling and eating disorders

The incidence of eating disorders (EDs) is increasingly affecting a wide age range and all genders. Body image dissatisfaction, weight concerns and unhealthy weight management are very frequent even among so-called normal weight people [55]. Furthermore, the phenomenon of orthorexia nervosa (ON), characterized by an excessive preoccupation with eating healthy food, is now the focus of attention of scientific research, both because it can significantly impact quality of life and nutritional status, and because it can represent a risk factor for other major EDs (anorexia nervosa – AN—and bulimia nervosa – BN) [14].

Different researchers have mainly focused their attention on the association between nutritional information and/or calorie labeling, and the psychological phenotypes of consumers.

In Martinez et al. [56], and M.W. Seward et al. [57], the usage of nutritional information in food labels in a college context was perceived by students as potentially unveiling EDs, and it was thought that nutrition labeling interventions may as well favor the evolution toward an ED or "feed" a disordered eating behavior, increasing the risk of exacerbating EDs and making recovering more difficult.

Different studies have raised concerns about the possibility that obesity prevention programs may increase the risk of developing EDs or exacerbate them due to negative effects on eating behaviors or on the emotional state of people at risk for disordered eating [56, 58–61].

In Roberto et al. [62] 92% of the sample, including self-reported ED subjects, were in favor of menu labeling, but five years later, in Haynos et al. [63], the same authors showed that, stratified by ED, subjects diagnosed with AN and BN selected the menus with fewer calories, while persons with Binge Eating Disorder (BED) choose the menus with higher calories among the labeled ones. In Lillico et al. [64], no adverse outcomes were found for the at-risk population: calorie labels did not differentially affect ED subjects. In Seward et al. [65], 60% of the students surveyed generally supported traffic light labeling (TLL) in the menus although, when asked if TLLs increased the risk of developing an ED, 16% of the participants answered "yes" and 47% affirmed that TLLs could exacerbate existing EDs. Because of these inconclusive results, McGeown [66] suggested that approaches other than calorie labeling can better help obesity management strategies and educational initiatives, and that intuitive eating could be a potential alternative for promoting healthy eating behavior [67].

Larson et al. [68] analyzed the correlation between calorie information on restaurant menus and weight-related behaviors (788 men and 1042 women; mean age: 31.0 ± 1.6 years). Among individuals who noticed calorie information, 38.2% reported that the information given had not been relevant to make a choice about what to order. The others reported instead that they used menu labels to limit calories, and this was especially observed in correlation with binge eating among women and it was associated with more weight-related concerns, dieting, and unhealthy weight-control behaviors among both women and men. For this reason, to avoid the promotion of unhealthy restrictive eating patterns in college cafeterias, several college campuses do not display nutrition information on the menus [69].

In another study published by Christoph et al. [70], it was showed that the use of nutrition facts was associated with a 23% and 10% greater likelihood of engaging in healthy and unhealthy weight control behaviors respectively, and with a 17% greater chance of engaging in binge eating. In men, it was associated with a 27% and 17% greater likelihood of engaging in healthy and unhealthy weight control behavior respectively, and with a lower level of intuitive eating [71]. Greater use of food labels was also associated with body weight control [71], and the propensity toward using nutrition facts was associated in women with a greater likelihood of engaging in BED [70], an ED that has been positively associated with disinhibition [72]. Restrained eating and food label use were found positively associated, while disinhibition and susceptibility to hunger were both positively, albeit weakly, associated with general food label use, suggesting that individuals presenting a higher level of disinhibition or susceptibility to hunger may use food labels to select food products that seem healthier or lower in fat or in energy to compensate for their overeating tendencies [73].

Different studies have highlighted that, when making hypothetical food choices from a menu that includes information about calories, individuals with AN and BN are more likely to order food with significantly fewer calories, whereas people with BED are more likely to order food with significantly more calories. More in general there is evidence that individuals with weight concerns and EDs may be particularly influenced by exposure to menu labels [63, 64, 74].

The role of nutrition information (and in particular the tendency of calories displaying) on eating behavior is therefore controversial. On the one hand, calorie labels on menus may negatively affect the eating or the psychosocial health of individuals with weight concerns; on the other hand, the provision of information may reduce feelings of anxiety when eating out among those who struggle with disordered eating [68].

Finally, the existing literature that examined the association between weight-related concerns in adults and the potential influence of menu labeling shows conflicting results [63, 64, 75].

### Negative and positive nutrition approach

All the aforementioned evidence highlights the significant limits of information based either on single nutrients or nutrients associated to calories. These limits might be even greater when talking about FOPNLs that suggest a negative relationship with specific nutrients and/or caloric intake.

Evidence is mixed about the efficacy of fear appeals and negative communication on driving people toward lasting lifestyle changes.

Even if not related to food, an example of a label that informs consumers about the risk of consumption are the cigarette warning labels (CWL). They have been largely implemented in the early 2000’s and their effects have been studied. Looking at the evolution of CWL’s effectiveness might be of interest to have an idea of possible long-lasting effects of a negative approach. For instance, studies on CWLs suggest that sizable proportions of adolescent smokers are not seeing, reading, or remembering CWLs. In addition, the knowledge of CWLs on packages and advertisement is not associated with reduced smoking [76]. The effectiveness of graphic warnings against cigarette smoking are mainly based on emotional responses and projections from simulation models, not necessarily leading to smoking cessation: while it seems this could affect smokers with lower level of nicotine dependence, there seems to be no effect on smokers with higher levels of dependence [77]. Effects also show cross-cultural differences, which highlights the importance of considering different messages based on the group being targeted [78]. Additionally, many studies analyze CWLs and fear appeals in relation to quitting smoking, but less is known about the role of fear appeals in persuading nonsmokers to avoid starting smoking.

Lastly, these campaigns revealed a wear-out effect tendency (decreasing effectiveness of a warning message over time). When textual CWLs have been introduced on tobacco product packages, after a first success they showed a wear-out effect. That led to an implementation of pictorial CWLs in addition to the text for a more effective outcome. Even so, after a prompt increase in effectiveness, the wear-out effect was observed again [79, 80] and because of that, some governments decided to change the displayed images frequently to maintain low- and middle results over time [74].

Besides the example of cigarettes, behaviors that lead to healthy habits sometimes seem to respond to negative messages [81, 82], but sometimes they respond to the opposite, with the best translation of intentions to live healthily into behavior associated with positive feelings, cognitive efforts and self-efficacy instead of fear arousal [83–85].

While in some areas, like cancer prevention, this kind of communication might work [86], the case of vaccine hesitancy has shown different results: the best approach to prevent it seems to be a multi-component one, where education material is developed, dialog-based communication strategies are incorporated, and literacy and critical thinking skills are improved [87].

It is worth mentioning that in recent years questions have been raised about scientific communication done so far, and since this is deeply connected with political choices in many fields (health included), it probably makes sense to ask whether it is not the case to redefine science communication and investigate its fundamentals, gaps, and areas of improvement, before moving on and making widespread decisions. For example, it seems that some scientists often see the public audience “as an ignorant, homogeneous group” [88]. This model of thinking could take paternalistic and dangerous turns at worst, and at best not conducive to an efficient communication and involvement. As in Simis et al. [88], “Only by filling this knowledge void and ridding people of their ignorance will the public be able to see the world in the same way as scientists”.

This has an understandable appeal for policymakers because it is a problem with a simple root (ignorance) and a simple solution. Simis et al. name this solution a “public-oriented education”, but in our specific case it would come down to labels, since talking about education in our instance would already be a step forward. It is probably worth asking if we do not need a double kind of education: one for the consumers, and one for the scientists and policymakers, to engage community members around issues that affect their health. A positive communication, positive feelings about the goals and coping techniques aimed at increasing response efficacy and self-efficacy, all seem to be crucial in promoting health-conscious actions, rather than a communication based on threatening health information [84, 85].

An example of negative approach was implemented in Chile in 2016 where a series of Warning Labels were applied on unhealthy foods and beverages to inform consumers about the high content in energy, sugars, SFAs and sodium, to restrict the marketing of these foods to children, and to restrict sales of these products in schools. Children’s households’ purchases were monitored and analyzed pre- and post-policy introduction to estimate the effectiveness of the intervention. Data show a decreasing in all the parameters observed (total energy, SFAs, sugars and sodium), but the reduction appears actually small: 16.4 kcal/capita/day for total energy, 11.5 kcal/capita/day for sugar, 2.2 kcal/capita/day for SFAs, and 27.7 mg/capita/day for sodium, combining the reductions observed both in beverages and in foods. [89].

Thus, it seems worth asking: "is it strategically appropriate to propose a “negative nutrition” through a FOPNL, which almost prohibits the consumption of certain foods, or would it not be better to work through a positive / proactive attitude?". In fact, the red signal on the food package does not give any information regarding the nutritional qualities of the food. It merely warns, based on the concentration of some nutrients considered harmful, without adequately promoting the nutrients and bioactive substances that might have a positive effect. Negative messages either are directed toward specialized audiences with detailed knowledge of the subject, or these “loss-framed messages” could leave consumers with a negative attitude and an unstable feeling: they could learn what not to do, but they do not learn how to change the behavior.

On the other hand, the positive approach, e.g., by suggesting the advisability of “eating five portions per day of seasonal, locally produced and possibly 5-color ranging fruit and vegetables”, not only describes the relevant nutritional aspects (i.e., fiber content, bioactive compounds etc.) but also the agri-food history of mostly plant-based dietary patterns, the promotion of territories and professions dedicated to traditional crops, the environmental sustainability that improves with plant-based foods, and the augmented sensory qualities of colors and flavors that come from a variety of choices: it concerns the socio-cultural, economic and sustainability values that characterize our diets, involving not only the foods that have made the food history of a community, but also conviviality, gastronomic practices, moderation in consumption, and food choices that take account of seasonality and local production.

On this basis, the Nordic Keyhole logo was developed and adopted in 2013 by five north-European countries (Iceland, Norway, Sweden, Denmark, Finland) to encourage customers to choose healthier foods that are typical of the Nordic Diet. Data from the most recent survey in 2017 found that 60% of consumers considered the Keyhole a good labeling system, showing this positive label to be reliable and used over time, with a small wear-out effect. Nonetheless, the three studies that assessed the impact of Nordic Keyhole and other FOPNLs on actual dietary intakes, found them to have minimal impacts on energy and nutrient intakes, but with some improvements in fat or fibers intakes observed for some population groups [10].

A case study from the PREDIMED cohort in Spain sees walnuts as an example of the importance of communicating about foods appropriately. Adding walnuts to the daily diet may have multiple health benefits, but only within the context of an overall healthful dietary pattern: it is therefore better to convey the role of walnuts in a plant-based diet rather than focusing only on them [90].

The GBD 2017 Diet Collaborators [49] found that suboptimal diet was responsible for more deaths than any other risks globally and that although sodium, sugar, and fat have been the main focus of diet policy debate in the previous two decades, the leading dietary risk factors for mortality were diets high in sodium, low in whole grains, low in fruit, low in nuts and seeds, low in vegetables, and low in omega-3 fatty acids. This finding suggests that dietary policies focusing on promoting the intake of components of diet for which current intake is less than the optimal level (positive nutrition) might have a greater effect than policies only targeting sugar and fat (negative nutrition).

A labeling system with a positive character that incorporates more information and education, avoiding judgments about what consumers are eating (e.g., red lights) may be more appropriate for promoting a healthy and sustainable diet [57]. Focusing on positive “to-do” rather than “not-to-do” behaviors should help more people adopt healthier eating habits [91]. Positive and gain-framed messages provide a kind of message that could be successful with the general population, which probably has limited awareness of the topic of the message, leaving a favorable impression and a committed, driven attitude [92, 93].

For example, an experimental study in Belgium exposed consumers to either a benefit-only message (about fish favorable lipid fraction and vitamin D content), a risk-only message (about dioxin and methyl mercury contamination), or a balanced message about fish consumption: the benefit message increased consumers’ intention to eat fish by 21% as compared to current fish consumption, and the risk-only message led to an 8% lower behavioral intention. It seems therefore that there is a “ceiling effect” with products that are already perceived healthy (like fish) for which we can only reach a minimal further increase [38].

As stated by BC Johnston et al. “nutritional recommendations must, acknowledge the low-certainty evidence and avoid strong “just do it” recommendations that can, as evidenced by the many low-fat recommendations worldwide, be very misleading” [94].

Moreover, some theories suggest that we should consider at least 3 components that could explain the enablers and barriers between people and healthy eating, as well as consumers’ behavior: capability, opportunity and motivation [92]. This complex combination of habits, home environment and self-efficacy [95] should be considered when planning health policy tools that cannot be limited to actions to undertake in supermarkets, but probably need to expand widely and deeply in society.

Confirming the merits of a positive educational approach, a meta-analysis focused on the effect of educational campaigns in improving healthy eating behaviors showed good results in both chronic disease and hemodialysis patients [96, 97]. Using positive messages can be a strategy that reinforces specific nutrition-related practices or behaviors, changes habits that contribute to poor health and goes toward positive actions.

## Limits

We are aware of the limitations of this narrative review: since it tries to include several extensive topics related to labels and to consumers behavior, some studies may have been overlooked. Another limit is that during the writing of this review, no scale for its quality assessment was used.

## Conclusion

The purpose of Front-of-Pack labels is to help consumers modify their food choices and, furthermore, to promote a healthy diet. This intervention might be successful if it affects a wide number of determinants, such as biological, economic, physical, social, psychological and educational aspects. To understand this complex framework, some of these aspects (such as the purchasing behaviors patterns, the health and hedonic aspects as determinants of the propensity to buy, and the determinants that influence food choices and eating behaviors) were briefly presented in “Healthiness and hedonic aspects as determinants of propensity to buy”–“Nutrition labeling and eating disorders”. The framework is complex, and many mechanisms or interactions are not yet fully understood, making it difficult to standardize an effective intervention.

Moreover, the role of FOPLs should be embedded in a broader concept of well-being and food security, which is “a situation that exists when all people, at all times, have physical and economic access to sufficient, safe and nutritious food that meets their dietary needs and food preferences for an active and healthy life” [98]. To this effect, a health policy intervention should try to answer to all people’s needs and take into account food traditions, habits and sociocultural aspects related to local territories. Hence, we focused on the possible relations between labeling and eating disorder and on the negative and positive nutrition approaches for a successful solution to the problem.

Currently, it is mandatory to find new, effective tools to inform consumers about healthy choices and sustainable eating patterns that can prevent NCDs and obesity. While it is not easy to communicate this information through Back-of-Pack labels, a FOPNL system based on single nutrients or on single foods may not consider the complexity of dietary patterns and of the psychology of consumers.

Moreover, given the complexity of this matter, and the scarcity of studies about a possible correlation between nutritional information in food labels and EDs, and given that we cannot exclude that nutrition labeling interventions might favor the evolution toward an ED or their exacerbation, more studies in this area are needed.

All the aforementioned evidence underlines the significant limits of information based either on single nutrients or nutrients associated with calories, suggesting that dietary policies should focus on promoting healthy dietary patterns instead only targeting a few nutrients. These limits might be even greater when talking about FOPNLs that suggest a negative relationship with specific nutrients and/or caloric intake. Negative communication is not necessarily a successful tool to educate consumers, to dialog with them and improving critical thinking.

Therefore, as challenging as it is, a labeling system with a positive character that incorporates more nutrition information and education and avoids messaging connoting judgment about what consumers are eating (e.g., red lights or dichotomic messages), may be more appropriate for promoting a healthy and sustainable diet. More studies in the field of the determinants of food choices (and their interactions) are needed to effectively transform the food system. While politics and the industry are important stakeholders in the food system reform, it is the person and the consumer who must be put at the heart of the analysis in order for the measures taken to be truly effective.

What is already known on this subject?

- Nutrition labels can be useful to inform consumers about their food choices and help them make healthy choices.
- FOPNLs have different objectives and provide information with different complexity.
- To date, FOPNLs’ impact has been largely studied in virtual scenarios that give an idea of what dietary improvements could be achieved under ideal circumstances and with an average consumer, but such a consumer does not exist in real-life conditions.
- The risk of encouraging orthorexic or otherwise dysfunctional behaviors in the population groups most at risk of developing eating disorders is still under-researched.

What does this study add?

This work aims to highlight the complexity of the relationship between FOPNLs and multiple aspects involved in eating and purchasing behaviors, to give a wider perspective on the matter and to shed light on the aspects that are still under-researched, such as the impact on people suffering from eating disorders, since research to date has not sufficiently explored the question. Furthermore:

- Further research is needed to extend the findings collected so far and to investigate the representativeness of the food samples used in the studies with respect to all the products present at the point of purchase. It is also necessary to investigate the possible overlap of virtual studies with the real-life scenario, characterized by biases, subjective variables, and various determinants that influence food choices.
- Studies are needed on the effect of FOPNLs in relation to the degree of nutritional literacy of the various socio-demographic groups, also looking for the outcome in relation to pre-existing disparities related to health status.
- This paper also would like to underline that the presence of a FOPNL (especially a directive one) can make the product look healthier, or less healthy, in the eyes of the consumer, and this could lead people to consume more of the products considered particularly healthy, i.e., those with a green sticker or logo on the packaging.
- Possible wear-out effect on FOPNLs (either informative or directive labels) should be considered to prevent a decrease in label effectiveness.
